# A relational perspective on women’s empowerment: Intimate partner violence and empowerment among women entrepreneurs in Vietnam

**DOI:** 10.1111/bjso.12348

**Published:** 2019-10-30

**Authors:** Marloes Anne Huis, Nina Hansen, Robert Lensink, Sabine Otten

**Affiliations:** ^1^ Department of Social Psychology University of Groningen The Netherlands; ^2^ Department of Economics, Econometrics, and Finance University of Groningen The Netherlands; ^3^ Development Economics Group Wageningen University The Netherlands

**Keywords:** empowerment, financial intra‐household decision‐making, gender inequity, intimate partner violence, self‐esteem, women

## Abstract

Research has mainly studied women’s empowerment assessing personal (e.g., self‐esteem) or relational (e.g., decision‐making) empowerment indicators. Women are not isolated individuals; they are embedded in social relationships. This is especially relevant in more collectivist societies. The current research provides a relational perspective on how husbands may hamper women’s empowerment by inflicting intimate partner violence (IPV) assessing women’s self‐reported experience. We tested the link between self‐esteem and experienced IPV on financial intra‐household decision‐making power among women entrepreneurs (*N* = 1,347) in Northern Vietnam, a collectivistic society undergoing economic development. We report two measurement points. As expected, self‐esteem (and not IPV) was positively related to more power in intra‐household decision‐making on small expenditures, which are traditionally taken by women. However, IPV (and not self‐esteem) was related to less decision‐making power on larger expenditures, traditionally a domain outside women’s power. We test and discuss the directionality of the effects and stress the importance of considering women’s close relationship when investigating signs of women’s empowerment.

Around the world, women typically have less access to power than men. For example, globally 15 million girls of primary school age will never get the chance to attend primary school (5 million more than boys), women earn less than men (global gender pay gap is 23%), and one in five women and girls under the age of 50 reported experiencing physical and/or sexual violence by an intimate partner within a 12‐month period (UN Women, 2018). Together, these figures illustrate that gender inequity can still be observed in several aspects of daily life. Consequently, empowering women is recognized as an important development goal of the United Nations and a key outcome for many interventions (e.g., UN Women, 2015).

To date, women’s empowerment has been predominantly investigated by assessing women’s personal agency (e.g., Alvarez, van Leeuwen, Montenegro‐Montenegro, & van Vugt, 2018) or their perceived influence over financial decision‐making within the household (for overviews, see Duvendack, Palmer‐Jones, & Vaessen, 2014; Huis, Hansen, Otten, & Lensink, 2017). Previous research mainly studied women as *individual agents* and did *not* consider the social relationships they are embedded in. However, humans are relational beings (e.g., Fiske, 1992). Studying women as individuals separated from their social contexts neglects the social structures that influence women’s position (Grabe, Grose, & Dutt, 2015). To study signs of women’s empowerment, we focus on decision‐making within households. These decisions most commonly involve both spouses (Duvendack *et al.*, 2014). In the current study, we, therefore, focus on women’s close relationship, which can entail both interdependence and competition (Glick & Fiske, 1996).

The current research offers a relational perspective on women’s empowerment by investigating signs of women’s empowerment and the impact of their partner (operationalized with intimate partner violence [IPV]). We conducted this research with women microfinance entrepreneurs in Vietnam. Vietnam is a collectivist cultural context where social relations strongly affect people’s life and well‐being – although within cultural variation exists (e.g., Phạm, 2013). This implies an interdependent model of self and relationships (Markus & Kitayama, 1991). Traditional gender norms widely exist in Vietnam (Duvvury, Carney, & Nguyen, 2012). In societies in which violence may be socially accepted, the risk of experiencing IPV is greater (Jewkes, 2002). In the following, we first define women’s empowerment and introduce the importance of taking a relational perspective when studying signs of women’s empowerment. We study this by focusing on a potential barrier, the experience of IPV, which may hinder women in expressing signs of empowerment. In the current research, only women reported their experience and perceptions.

## Defining women’s empowerment

Women’s empowerment is defined as the process through which women acquire and use resources in an agentic manner to reach certain achievements, which have so far been denied to them (e.g., Kabeer, 1999). As such, women’s empowerment is a process from being disempowered to becoming empowered (e.g., Kabeer, 1999; Malhotra, Schuler, & Boender, 2002). In other words, empowerment enables people to act on and improve issues that are important for their individual lives, their communities, and society at large (e.g., Cattaneo & Chapman, 2010). Women’s empowerment has been operationalized with very different indicators to assess this broad concept. A recent literature review offers a framework to categorize the diverse measures of women’s empowerment in three distinct but related dimensions (Huis *et al.*, 2017) ranging from (1) personal empowerment such as self‐esteem (e.g., Kato & Kratzer, 2013), and (2) empowerment in relation to others, such as financial intra‐household decision‐making (e.g., Duvendack *et al.*, 2014), and (3) societal empowerment (e.g., representation of women in parliament).

It is important to stress that power is a social–relational concept (Thibaut & Kelley, 1959). In other words, an individual’s power can be understood only in relation to another individual or group. Research on power has shown that a personal sense of power, the belief that one can influence others, is dependent on specific relationships (Anderson, John, & Keltner, 2011). A person may be confident about their power and how they could influence one friend but less so regarding another friend. In the current research, we assess this belief as personal empowerment (i.e., self‐esteem). To investigate whether personal empowerment is related to the utterance of power, we studied intra‐household decision‐making power as an indicator of relational empowerment. This paper focusses on women’s personal and relational empowerment. To the best of our knowledge, research has not yet systematically investigated the directionality of the dimensions.

### Women’s empowerment at the personal level

Previous research assessed the impact of access to microfinance services on women’s empowerment with measures reflecting women’s perceptions of individual strength, such as control beliefs (e.g., Hansen, 2015) and self‐esteem (e.g., Kato & Kratzer, 2013). Research that has used this type of indicators mainly stressed women’s empowerment as a personal notion of being in power (e.g., Hansen, 2015). Moreover, self‐esteem is described as a fundamental principle of empowerment (Naz, 2006) and an important starting point before people can gain power to influence their own lives (e.g., Rowlands, 1998). While the universality of self‐enhancement has been questioned (e.g., Heine, 2005), other research supports the structural equivalence of self‐esteem across cultures, although the subcomponents (self‐liking and self‐competence) systematically vary across cultures (Schmitt & Allik, 2005). Research conducted in Vietnam investigated self‐esteem, which corresponded with the universal construct (Alessandri, Cenciotti, Łaguna, Różycka‐Tran, & Vecchione, 2017). We assessed women’s self‐esteem as one indicator of women’s personal empowerment.

### Women’s empowerment at the relational level

Women are embedded in different relations such as their partnership, family, or community, which can influence their personal behaviour. Social relationships are imperative for human beings worldwide and define who we are (e.g., van Zomeren, 2016). Relationships both within and outside the nuclear household are important to people’s sense of self (e.g., Markus & Kitayama, 1991) and can contribute to people’s feeling of empowerment (e.g., Stromquist, 2015). People, especially in cultural contexts adhering to an ecology of embedded interdependence, may even prioritize the well‐being of the group over their own well‐being (e.g., Kurtiş, Adams, & Estrada‐Villata, 2016). Moreover, the different social networks people belong to, such as family or community, influence how people should behave (i.e., deductive norms) and how people adhere to the social norms (i.e., inductive norms). These norms are central for these societies (e.g., Gelfand, Harrington, & Jackson, 2017). Vietnam can be characterized a patriarchal society in which people generally adhere to traditional gender norms where men are respected heads of families while women are responsible for maintaining a family’s harmony (Duvvury *et al.*, 2012).

Women’s close relationship may be the most defining social relationship in which women are embedded and which impacts on women’s empowerment (e.g., Belcher, Peckuonis, & Deforge, 2011). To date, research in the field of microfinance services has mainly studied intra‐household decision‐making power as an indicator of relational empowerment (Duvendack *et al.*, 2014). This scale assesses the extent to which women have a say in financial investments across different domains. In the current research, we focussed on financial intra‐household decision‐making power among couples to assess one aspect of women’s position in their close relationship.

### Intimate partner violence

Previous research showed that men’s controlling behaviour influenced different aspects of women’s empowerment. More precisely, husband’s controlling behaviour (e.g., allowing women to visit family and friends) was negatively related to self‐esteem and positively to physical violence and depression among women business owners in Tanzania (Dutt, Grabe, & Castro, 2016). Similar results were found among women landowners in Nicaragua and Tanzania showing that husbands controlling behaviour was positively related to women’s experience of physical and psychological violence (Grabe *et al.*, 2015).

The relation between women and men is defined by an unequal distribution of power (Pratto & Walker, 2004). According to the gendered power model, four forms of power constitute the relation between women and men. More precisely, men hold more power than women in terms of strength (e.g., physical and emotional violence, also called intimate partner violence), access to resources (e.g., financial resource control), social obligations (e.g., distribution of household responsibilities), and gender ideology (e.g., gender norms). Women may face different expressions of male domination within their close relationship, for example, through intimate partner violence or time‐consuming household work hindering women’s ability to develop and express feelings of empowerment (e.g., Pratto & Walker, 2004; Stromquist, 2015).

We conducted the current research in Vietnam where 27% of women indicated to have experienced physical, sexual, and/or emotional violence, in the past 12 months (Government of Viet Nam, 2010). Moreover, 58% of the women reported having experienced at least one of these three types of violence in their lifetime. Thus, intimate partner violence is a substantial problem for many Vietnamese women and may influence women’s expression of empowerment.

### The relation between self‐esteem, intimate partner violence, and intra‐household decision‐making

To the best of our knowledge, research has not yet systematically investigated the impact spouses may have on women’s empowerment. Previous research has examined links between women’s ownership of land and well‐being and men’s controlling behaviour and expression of IPV (e.g., Grabe *et al*., 2015). The dynamics determining women’s well‐being in social structures adhering to unequal gendered hierarchies are complex. This research offers a first attempt to unravel parts of this complexity by examining the relation between women’s personal sense of empowerment (i.e., self‐esteem), their expression of empowerment (i.e., intra‐household decision‐making on small and larger expenditures), and one potential relational barrier to women’s empowerment (i.e., intimate partner violence).

We assume that women first may need to develop a sense of personal empowerment before they can act out and express empowerment within their close relationship (see also capacity for action argument, Hansen, 2015).

Within the cultural context of the current study, Vietnam, women traditionally make decisions about small household expenditures, such as food (e.g., Johnson, 2016). In contrast, men traditionally make decisions about larger expenditures, such as buying and selling of land and property (e.g., Kabeer, 1999). Decisions that are conventionally not made by women may be most indicative of greater empowerment (e.g., Dutt *et al*., 2016; Johnson, 2016). Therefore, we distinguish between small and larger expenditures in our research.

#### Small expenditures

Previous research argues that women’s empowerment begins with changes in women’s personal agency beliefs to be able to influence decisions (Anderson *et al.*, 2011; Hansen, 2015; Kabeer, 2012). Peoples’ trust in their own abilities is an important factor determining behaviour required to achieve desired outcomes (e.g., Bandura, 1997). This trust in one’s own abilities may be especially relevant for decisions about expenditures that are traditionally taken by women. Thus, only self‐esteem and not IPV should be positively related to financial intra‐household decision‐making power on small expenditures among women entrepreneurs because men traditionally do not interfere in this domain (Hypothesis 1).

#### Larger expenditures

Men traditionally make investments in larger expenditures. If women gain power over these decisions, this is indicative of greater empowerment (e.g., Dutt *et al*., 2016; Johnson, 2016). However, men’s controlling behaviour over their wives is one of the most apparent barriers to women’s empowerment (Dutt *et al.*, 2016; Grabe *et al*, 2015; Kabeer, 2012). Thus, only IPV and not self‐esteem should be negatively related to financial intra‐household decision‐making power on larger expenditures among women entrepreneurs (Hypothesis 2).

## Current research context

The current research was conducted in Northern Vietnam among women who were all running an income generating activity and were members of a large microfinance institution. We selected this unique sample for two reasons. First, Vietnam is categorized as a lower‐middle‐income economy currently undergoing rapid economic development (The World Bank, 2014). Offering access to microfinance services is one approach to stimulate the economic development of the nation. These small‐ and medium‐sized businesses are important drivers for economic development (Edmiston, 2007). Importantly, women’s participation in microfinance programmes reduces credit constraints and may stimulate women’s agency. Women who join microfinance programmes start developing stronger feelings of empowerment (e.g., mobility, decision‐making, legal and political awareness) compared to women who do not join these programmes (Hashemi, Schuler, & Riley, 1996; for an introduction to microfinance and empowerment see Hansen, Huis, & Lensink, in press). Women’s participation in these programmes is likely to strengthen their financial skills and stimulate change in the household power balance. Women’s participation in nondomestic roles and earning of income may stimulate a shift in existing gendered labour divisions within the household towards more egalitarian roles (Wood & Eagly, 2012). Thus, the entrepreneurs participating in our research should show first signs of empowerment and be an interesting group to test our assumptions.

Second, in Vietnam, people generally adhere to a cultural ecology of embedded interdependence. Relationships with others – especially the family – strongly influence peoples’ lives (e.g., Phạm, 2013). Gender roles portraying women as the primary caregivers and men as primary breadwinners influence daily life (e.g., Duvvury *et al.*, 2012), and women are perceived as inferior to men according to Confucian‐based traditional values and beliefs (e.g., Walker, & Truong, 2016). Although microfinance programmes are implemented with the intention to strengthen women’s empowerment and support them, some researchers found negative side effects such as men’s controlling behaviour over their wives (e.g., Dutt *et al.*, 2016) and an increase in domestic violence among women who participated in such programmes (e.g., Goetz & Gupta, 1996). In sum, this sample provides a unique chance to test the relation between IPV and indicators of women’s empowerment in a cultural context, where people commonly have an interdependent model of self and relationships (e.g., Markus & Kitayama, 1991).

## Method

### Sample

We interviewed 1,351 women who were members of the largest microfinance institution in Northern Vietnam, the Tao Yeu May fund (TYM) in 2015. The same 1,347 women entrepreneurs were interviewed again in 2016; only four women did not participate again. We report analyses of the merged sample of 1,347 women entrepreneurs. We obtained ethical approval for this research from the Ethical Committee Psychology of the University of Groningen, The Netherlands. The women borrowers in our sample were members of 86 different lending centres in Vinh Phúc and Hà Nội. The microfinance institute started operating in 1992 and has since granted 98,623 loans to women borrowers. Women can receive small loans ($43 to $1,000) to develop their income generating activity. At time 1, the women in this study had been members of the microfinance institution for on average 7.9 years (*SD* = 4.14, range = 0–23 years) and most women (74.94%) were granted a microloan at the time of the study. 70% of the women managed a farming activity, such as growing rice or flower cultivation and 30% ran a small business, such as a retail shop, or worked in manufacturing, such as dress making. All participants were married or cohabiting. The average age was 45 years (*SD = *9.85*,* range: 22–73). The majority of the women completed secondary school or higher (78.92%) and lived with on average five persons in their household including themselves (*M* = 4.79; *SD = *1.49*,* range: 1–14).

### Procedure

The present study is part of a larger project examining the impact of a microfinance programme (see Bulte, Lensink, & Vu, 2017; Huis, Lensink, Vu, & Hansen, 2019). Native enumerators were intensively trained and conducted one‐to‐one interviews in Vietnamese. We used interviews based on a quantitative survey because some women were not used to filling in questionnaires (e.g., see also Van de Vijver & Leung, 1997) and to increase the response rate. Women were asked questions about demographical information, their economic situation, self‐esteem, time preferences, financial intra‐household decision‐making power, business practices and knowledge, IPV, and lending centre characteristics. In this study, we focus on self‐esteem, IPV, and financial intra‐household decision‐making. On average, the interviews lasted 1 hr (ranging from 45 to 90 min).

### Measures

#### Self‐esteem

We assessed self‐esteem as an indicator of women’s personal empowerment. The original scale (Rosenberg, 1965) consisted of five positively (e.g., ‘On the whole, I am satisfied with myself’) and five negatively framed items (e.g., ‘I feel I do not have much to be proud of’). One original negatively framed item had a positive meaning due to the translation in this cultural context (‘I wish I could have more respect for myself’). After discussing this issue with a native speaker, we coded this item as a positively framed item. Thus, we assessed self‐esteem with six positively and four negatively framed items. Women were asked to indicate to what extent they agreed or disagreed with each statement on a 5‐point scale ranging from strongly disagree (1) to strongly agree (5). We report our findings based on the full 10‐item self‐esteem scale which showed a rather low reliability (see Table 1; see Schmitt & Allik, 2005, for similar low levels of reliability in collectivistic societies) and was not measurement invariant (see supplementary materials in the Appendix S1, p. 44). These findings are very similar to the results observed when only including the six positively phrased items.

**Table 1:** Descriptive overview of and correlations between the study variables at time 1 and time 2

Variable	*M* Time 1	*M* Time 2	*t*‐test	1.	2.	3.	4.
1. Self‐esteem	3.70 (.38) [α = .66]	3.63 (.48) [α = .78]	−1.68	–	.16***	.17***	.04^−1^
2. Dummy IPV	0.46 (.50) [α = .50]	0.60 (.49) [α = .77]	4.30***	−.08**	–	−.02	−.17***
3. Decision‐making small expenditures	2.49 (.66) [α = .82]	2.41 (.69) [α = .66]	−2.16*	.32***	−.09**	–	.24***
4. Decision‐making larger expenditures	3.76 (1.06) [α = .77]	3.50 (1.42) [α = .81]	−2.62*	.04	−.28***	.24***	–

Notes

The means and standard deviations – in brackets – are reported for all study variables at time 1 and time 2. Cronbach’s alpha is reported for each scale at both times in square brackets. The alpha for Dummy_IPV refers to the reliability of the seven included act of IPV. The *t*‐test reports the change over time from time 1 to time 2. The correlation table reports correlations of the study variables at time 1 above the diagonal and the correlations of the study variables at time 2 below the diagonal.

* *p* < .05; **p < .01; ***p < .001.

#### Intimate partner violence

Intimate partner violence includes various types of abuse inflicted by an intimate romantic partner (e.g., McCloskey, 2007). We included a broad measure of IPV encompassing different types of acts including ‘emotional violence’ (example item: insisted on knowing where you are at all times) and ‘physical violence’ (example item: pushed, slapped, beat, or hit with a fist; adapted from Straus, 1979; World Health Organization, 2005; all items, see Table 2). Women were asked to indicate how often, in the previous 6 months, their spouse inflicted seven types of IPV on a 5‐point scale, ranging from 0 (never) to 4 (very often). Based on previous research (e.g., Withaker, Heileyesus, Swahn, & Saltzman, 2007), we decided to focus on the experience of violence *per se* and not the frequency of violence (see Table 2 for the frequency of experienced IPV). We computed a dummy variable to assess whether women had experienced at least one or more acts of IPV in the last 6 months (= 1) or had not experienced any act of violence (= 0). This dichotomization best represents our data because women generally indicated that acts of IPV either were not present or were rarely present (see Table 2). This measure encompasses an indication of the presence of IPV also for women who *only* experienced IPV rarely.

**Table 2:** Items measuring intimate partner violence at time 1 and time 2

Intimate partner violence items	Reported frequency of act time 1					Reported frequency of act time 2					
	Never (%)	Rarely (%)	Sometimes (%)	Often (%)	Very often (%)	Never (%)	Rarely (%)	Sometimes (%)	Often (%)	Very often (%)
Verbal aggression	58.05	33.93	7.80	0.15	0.07	47.73	43.03	9.02	0.23	–
Physical assault (pushed, slapped, beat, or hit with a fist)	91.24	8.54	0.22	–	–	79.62	16.89	3.48	–	–
Threatened and used with an object like sticks, knife, etc.	99.55	0.30	0.15	–	–	92.65	4.09	2.12	1.14	–
Kept you from seeing your family members or friends	99.78	0.22	–	–	–	96.25	2.85	0.49	0.33	0.08
Insisted on knowing where you are at all times	99.26	0.67	0.07	–	–	90.73	6.31	2.51	0.46	–
Wanted you to ask permission before doing anything	94.73	3.41	1.19	0.67	–	79.44	17.98	2.43	0.15	–
Insulted or humiliated you in front of other people	87.75	10.02	1.48	0.74	–	75.04	18.13	6.53	0.30	–

#### Intra‐household decision‐making

We assessed intra‐household decision‐making with a standardized scale (household decision‐making index; Mizan, 1993), which is a commonly used proxy to assess women’s empowerment (for a review see Duvendack *et al.*, 2014). We selected 11 suitable items to assess who usually takes specific financial decisions within the household. Women were asked to indicate who in their household usually takes each of the 11 different financial decisions: their husband alone (0), they and their husband together (0.5), or they alone (1) (see also Huis, Lensink, Vu, & Hansen, 2019). We summed up the values to create an index. Based on our theoretical reasoning above and previous research (e.g., Johnson, 2016), we conducted a confirmatory factor analysis showing that the 11 items loaded on two separate factors, namely small expenditures and larger expenditures (see Table A1 in the Appendix S1 for an overview of the items, factor loadings, and explained variance).1Ideally, we would have included an equal number of small and larger expenditure decisions. However, there are only a few domains in which women traditionally decide alone.
*Small expenditure decision‐making* was assessed with three items related to the daily domain, such as food and clothing (e.g., ‘Who makes most decisions about what food items to purchase?’). The final scale ranged from 0 (= woman made no decisions) to 3 (= woman made all three decisions alone). In line with our assumption that women’s say about small expenditures should not change over time, this scale showed measurement invariance over time (see supplementary material in Appendix S1, p. 44). *Larger expenditure decision‐making* was assessed with eight items related to larger expenditures for which men are traditionally in charge, such as loans, savings, and investments (e.g., ‘Who makes most decisions about asking for a loan?’)*.* The final scale ranged from 0 (= woman made no decisions) to 8 (= woman made all eight decisions alone).

## Results

Table 1 provides an overview of the means, standard deviations, alphas, and correlations of all study variables. Women expressed relatively high levels of self‐esteem (*M*_Time1_ = 3.70, *SD* = 0.38; *M*_Time2_ = 3.63, *SD* = 0.48) showing no change over time *t*(2,686) = −1.68, *p *= .097, *d* = 0.16. In total, 45.66% (time 1; *n* = 615) and 60.28 % (time 2; *n* = 812) of the women indicated that they have at least experienced one of the seven acts of IPV in the last 6 months. This is an increase of almost 15 %, a significantly small increase over time *t*(2,694) = 4.30, *p *< .001, *d* = 0.16. Furthermore, the majority of women took financial decisions on small expenditures alone (*M*_Time1_ = 2.49, *SD* = 0.66; *M*_Time2_ = 2.41, *SD* = 0.69), showing a significantly small decrease over time *t*(2,682) = −2.16, *p* = .034, *d* = 0.12. Women took less financial decisions on larger expenditures alone, which they more commonly take together (*M*_Time1_ = 3.76, *SD* = 1.06; *M*_Time2_ = 3.50, *SD* = 1.42) showing a significantly small decrease over time *t*(2,598) = −2.62, *p* = .01, *d* = 0.30. For details, see Table 3.

**Table 3:** Frequency table for division of financial decision‐making in the household at time 1 and time 2

Overall intra‐household decision‐making	Time 1			Time 2		
	Husband alone (%)	Couple joint (%)	Wife alone (%)	Husband alone (%)	Couple joint (%)	Wife alone (%)
Larger expenditure decision‐making						
… asking for a loan?	5.0	89.0	6.0	15.9	69.7	14.4
… consumer durable items?	16.0	77.2	6.8	30.0	57.0	13.0
… what health expenditures to make?	3.4	56.2	40.3	10.2	52.9	36.9
… saving for business and for household?	5.5	67.5	27.0	15.9	62.8	21.3
… expenses for home purchase, improvement, or repair?	20.0	74.1	5.9	32.4	54.4	13.2
… where to invest surplus money?	8.5	64.8	26.7	14.2	59.9	25.9
… how to assist family members?	9.9	82.4	7.8	19.3	67.3	13.4
… saving for household?	6.9	68.6	24.5	11.1	61.7	27.1
Small expenditure decision‐making						
… what food items to purchase?	1.8	23.1	75.1	4.4	18.9	76.7
… what educational expenditures to make	2.7	41.0	56.4	7.1	38.4	54.6
… what clothing items to purchase?	2.5	23.9	73.6	7.7	22.6	69.7

**Figure 1:**
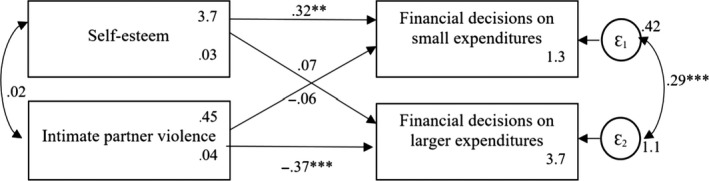
Conceptual model predicting women’s financial decision‐making on small and larger expenditures at time 1. Means and robust standard errors are reported for the endogenous variables. *p < .05; **p < .01; ***p < .001.

**Figure 2:**
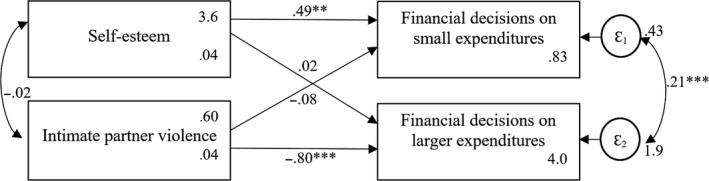
Conceptual model predicting women’s financial decision‐making on small and larger expenditures at time 2. Means and robust standard errors are reported for the endogenous variables. *p < .05; **p < .01; ***p < .001.

**Figure 3:**
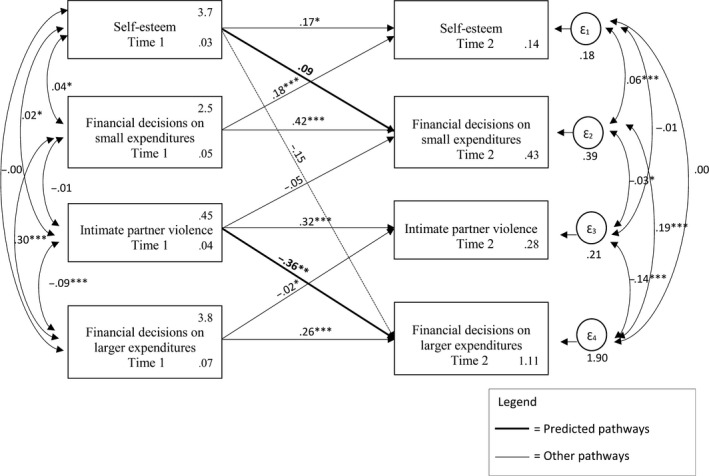
Cross‐lagged conceptual model testing the dynamic relationship of all study variables at time 1 and time 2. Means and robust standard errors are reported for the endogenous variables. *p < .05; **p < .01; ***p < .001.

**Figure 4:**
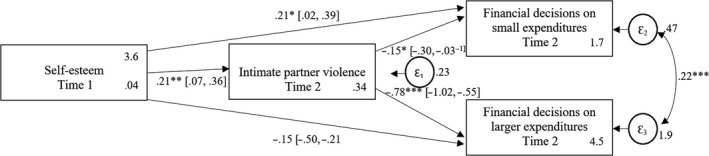
Mediation model predicting women’s financial decision‐making on small and larger expenditures at time 2 by self‐esteem at time 1 mediated by experienced intimate partner violence at time 2. Means and robust standard errors are reported for the endogenous variables. The confidence intervals for all coefficients are in box brackets. The indirect effect of self‐esteem at time 1 via IPV at time 2 on small expenditures at time 2 is not significant −.03 (−0.07, 0.00). The indirect effect of self‐esteem at time 1 via IPV at time 2 on larger expenditures at time 2 is significant −.17 (−0.29, −0.04). *p < .05; **p < .01; ***p < .001.

**Figure 5:**
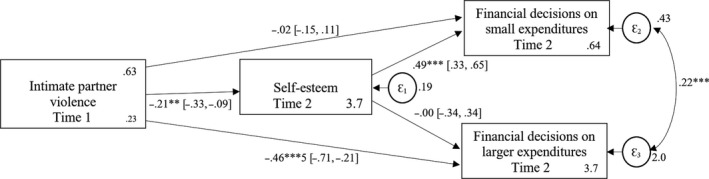
Mediation model predicting women’s financial decision‐making on small and larger expenditures at time 2 by experienced intimate partner violence at time 1 mediated by self‐esteem at time 2. Means and robust standard errors are reported for the endogenous variables. The confidence intervals for all coefficients are in box brackets. The indirect effect of IPV at time 1 via self‐esteem at time 2 on small expenditures at time 2 is significant −.10 (−0.18, −0.03). The indirect effect of IPV at time 1 via self‐esteem at time 2 on larger expenditures at time 2 is not significant 0.00 (−0.07, 0.07). *p < .05; **p < .01; ***p < .001.

We first controlled for age and educational level in all analyses. Both variables were not related to any of the study variables (all *p*s > .17). Therefore, we decided to report all further analyses without controlling for both variables (see Figure A3 in the Appendix S1 for the conceptual model with age and educational level included). We used Stata (2017) to analyse our data. As our data set has a nested structure (women receiving loans in 86 different lending centres), we clustered the standard errors at the lending centre level to control for possible dependency between women borrowers receiving microloans in the same lending centres for all analyses. Next, to be able to draw conclusions about the relative importance of the included predictors at the two measurement points, we used structural equation modelling to test the conceptual model depicted in Figures 1 and 2. Next, we examined a cross‐lagged analysis to explore directionality in the conceptual model (Figure 3). Lastly, we tested two possible underlying processes: mediating self‐esteem at time 1 by experience of IPV at time 2 (see Figure 4) or vice versa (see Figure 5) on women’s financial decision‐making on small and larger expenditures at time 2. We report these results in the text below.

### Conceptual model

The conceptual model included reported self‐esteem and experienced IPV as exogenous variables (see Figures 1 and 2). Direct paths from these exogenous variables to financial decision‐making on small and on larger expenditures were included. We expected that women’s self‐esteem, but not IPV, should predict financial decision‐making on small expenditures. Furthermore, we expected that IPV, but not self‐esteem, should predict financial decision‐making on larger expenditures. The model included the covariance between the two exogenous variables and the error terms of the endogenous variables.

The conceptual model fits the data adequately at both measurement points: time 1: CFI = 1.00, TLI = 1.00, RMSEA = .000, 90% CI (0.000, 0.000), SRMR = .000; time 2: CFI = 1.00, TLI = 1.00, RMSEA = .000, 90% CI (0.000, 0.000), SRMR = .000, but explained more variance at time 2 (CD = .170) than at time 1 (CD = .069).2We first analysed the data using clustered standard errors to control for possible dependency within lending centres, which only offers SRMR and CD as fit indices. We next conducted the analyses without clustered standard errors, which offer more fit indices. The results were the same. Thus, we report model fit indices based on the models without clustered standard errors (for a discussion on the use of fit indices for models with nested data, see e.g., Chen, Luo, Palardy, Glaman, & McEnturff, 2017). As expected, women’s self‐esteem was positively related to women’s financial decision‐making on small expenditures (*B*_Time1_ = .32[.11], *p* = .003; *B*_Time2_ = .49[.08], *p* < .001), whereas IPV was not related to it (*B*_Time1_ = −.08[.09], *p* = .490; *B*_Time2_ = −.08[.06], *p* = .246). Importantly, IPV was negatively related to women’s financial decision‐making on larger expenditures (*B*_Time1_ = −.37[.09], *p* < .001; *B*_Time2_ = −.80[.13], *p* < .001), whereas self‐esteem was not related to it (*B*_Time1_ = .07[.15], *p* = .642; *B*_Time2_ = −.02[.17], *p* = .894). Results of two measurement points provide first evidence for the robustness of our conceptual model.

Next, we tested a cross‐lagged analysis to examine directionality between the variables in the conceptual model. We included all variables at time 1 and at time 2 and examined the expected relations, reversed relations, unexpected relations, and the stability of our constructs (see Figure 3). The conceptual model did not fit the data adequately: CFI = .543, TLI = −.189, RMSEA = .237, 90% CI (0.226, 0.247), SRMR = .051, but explained more variance (CD = .339) than both models examining the relation between the variables at one time point (Figures 1 and 2). Interestingly, we observe the same general pattern of results.

#### Mediation analyses

Lastly, we estimated two possible underlying pathways. First, we estimated women’s financial intra‐household decision‐making on small and on larger expenditures at time 2 by self‐esteem at time 1 mediated by IPV at time 2 (Figure 4) showing an adequate fit, CFI = 1.00, TLI = 1.00, RMSEA = .000, 90% CI (0.000, 0.000), SRMR = .000, CD = .044. However, the relationship between self‐esteem at time 1 and women’s decision‐making on small expenditures at time 2 was not mediated by IPV at time 2 (see also Figure A1 in the Appendix S1 for an illustration).

Second, we estimated women’s financial decision‐making on small and on larger expenditures at time 2 by IPV at time 1 mediated by self‐esteem at time 2 (Figure 5) showing an adequate fit, CFI = 1.00, TLI = 1.00, RMSEA = .000, 90% CI (0.000, 0.000), SRMR = .000, CD = .078. However, the relationship between IPV at time 1 and women’s decision‐making on larger expenditures at time 2 was not mediated by self‐esteem at time 2 (see also Figure A2 in the Appendix S1 for an illustration).

## Discussion

This research offers a relational perspective on understanding women’s empowerment among women entrepreneurs in Northern Vietnam. We interviewed women twice, 1 year apart and checked the robustness of our conceptual model. We find the expected pattern of results at both measurement points as well as partly in a cross‐lagged analysis (IPV at time 1 and not self‐esteem decreased decision‐making power on larger expenditures at time 2). In general, our results show that women’s self‐esteem (at the same time point), but not IPV, is positively related to intra‐household decision‐making power on small expenditures. This finding supports previous research stressing the importance of personal capacity beliefs to achieve desired outcomes (e.g., Hansen, 2015; Kabeer, 2012). However, we have to interpret these effects with caution due to the low reliability and measurement invariance of the self‐esteem scale. More importantly, this picture changes when focusing on decision‐making in domains that are traditionally outside women’s power, namely investments about larger expenditures within a household. In this case, IPV but not self‐esteem was negatively related to intra‐household decision‐making power on larger expenditures. IPV against women may hinder women in having a say over household spending on larger expenditures. Although women may show signs of personal empowerment, a bad relationship with their spouse may hinder women to express empowerment on other dimensions (e.g., Anderson *et al.*, 2011; Pratto, 2016). Interestingly, self‐esteem did not change over time. However, we found a small effect for an increase in IPV over time and a decrease in women’s decision‐making power with respect to small and larger expenditures over time. We can only speculate about these results. Previous research shows that women borrowers may experience more IPV compared to non‐borrowers (Rahman, Hoque, & Makinoda, 2011). Some men may exert IPV to regain greater control that stems from the breaking of traditional gender roles (for a discussion, see Dutt *et al.*, 2016; Huis, Hansen, Otten, & Lensink, 2019). Furthermore, giving women a financial resource such as microloan may or may not enable them to have a stronger say with respect to financial decision‐making. Overall, consistent evidence for the effect of microcredit on women’s power on financial household expenditures has not been found (see for a systematic review of the evidence Vaessen *et al.*, 2014) Importantly, the mean differences signal that relations are dynamic and change over time (for an overview see Schoebi & Randall, 2015). This is further supported by a recent meta‐analysis showing that people’s self‐esteem and social relationships reciprocally influence each other over time (Harris & Orth, 2019).

To gain first insights in the dynamics of our study variables, we tested two possible underlying pathways, which may explain women’s say in financial intra‐household decision‐making. Neither self‐esteem nor IPV mediated the predicted effects. Our study design does not allow us to draw causal conclusions about the directionality of the effect. However, our robust findings seem to suggest that self‐esteem and IPV have different effects on two different types of decision‐making.

Women’s decision‐making power over larger expenditures is more indicative of greater empowerment (e.g., Dutt *et al*., 2016; Johnson, 2016). Whereas women’s involvement in a market economy (here microfinance) may stimulate the development of more individual independence, relational interdependence remains important (for a similar argument see e.g., Kağitçibaşi, 2005; Manago, 2014). We suggest that women’s close relationship may be one important factor to consider when studying women’s empowerment (see also Kurtiş *et al*., 2016). In line with a recent report (Picon *et al.*, 2017), we argue that reducing intimate partner violence should in the long‐run increase women’s empowerment. Improving relationships towards more mutual trust and less partner violence may empower women to have a say in their social life (e.g., Christens, 2012).

In the current research, we focused on one important relationship, women’s close relationship. However, other interpersonal relations such as kinship or community are also relevant to women’s sense of self and offer shared values and norms (e.g., Anderson, Adams, & Plaut, 2008). In fact, people living in some societies may give priority to kinship over their marital partner (e.g., Salter & Adams, 2012). Participants in our research were all women entrepreneurs and members of a microfinance institution. Women entrepreneurs commonly run a joint family business together with their spouse and are jointly involved in the intra‐household decision‐making. Our findings suggest that this close relationship is important in women’s ability to express signs of empowerment.

In general, power barriers and prescribed gender roles often inhibit women’s abilities to develop stronger feelings of empowerment (e.g., Connell, 1987; Pratto & Walker, 2004). A recent meta‐analysis suggests that addressing gendered power imbalances and traditional gender roles next to providing microfinance services is needed to strengthen women’s position (for a broader discussion see Duvendack & Mader, 2019). Our research provides additional evidence for the importance of considering the broader context, in which women are embedded. Importantly, controlling behaviour by partners may not only hinder women’s decision‐making power in this specific context but also play an important role in women’s expression of empowerment in other cultures as well.

Notably, previous research showed that women microfinance borrowers were more empowered (e.g., higher self‐esteem, stronger decision‐making power) compared to non‐members (e.g., Hashemi *et al*., 1996). This suggests that our findings might be even more pronounced in a sample of women who do not seek microfinance services suggesting that we may offer a more conservative test of our hypotheses.

### Limitations and future research

There are three important limitations of our work. First, our study design only offers limited insights in the underlying dynamics of the development of women’s empowerment. Regarding women’s self‐esteem, we consider the suggested directionality from higher self‐esteem to more financial decision‐making power more likely than the reverse order, although our cross‐lagged analysis does not substantiate this expectation. The expected directionality is in line with theorizing on power; people’s sense of power should influence people’s expression of power (see Anderson *et al.*, 2011). Indeed, self‐esteem is a personality characteristic that is relatively stable across time and contexts for adults (e.g., Orth, Trzesniewski, & Robins, 2010), even though the relation between people’s self‐esteem and their social relations may be dynamic and reciprocal (Harris & Orth, 2019). The directionality between IPV and having financial intra‐household decision‐making power is less straightforward. Some men may exert controlling behaviour or IPV over their wives to regain greater decision‐making power that stems from the breaking of traditional gender roles (for a discussion, see Dutt *et al.*, 2016). Research on close relationships shows that intimate relationships are dynamic and may change over time (for a literature review, see Schoebi & Randall, 2015; complexity of co‐regulation in interpersonal relations, see Butler, 2015). Accordingly, longitudinal research is needed to substantiate the directionality and dynamics.

Second, we aimed to use standardized scales to investigate our hypotheses. We selected three indicators for our research, namely self‐esteem, financial intra‐household decision‐making, and IPV. The studied phenomena are rather complex and do not only include the three selected aspects. For example, financial intra‐household decision‐making is also dependent on other factors such as the financial situation of a household and the personalities of the couple. Therefore, our results only explain relatively little variance. Future research is needed to test our assumptions with additional indicators of personal (e.g., autonomy, agency) and relational (e.g., household obligations) empowerment and structural dependency (e.g., control over resources, earnings potential). Future research should employ cultural‐sensitive measures for especially personal empowerment. In the current research, self‐esteem had a low reliability (especially at time 1) and did not show measurement invariance. Thus, the effects have to be interpreted with caution and may signal that self‐esteem may not be a clear concept in this cultural context. People in more collectivist cultures tend to evaluate themselves less in terms of self‐enhancement (e.g., Heine, 2005; Heine & Hamamura, 2007).

Third, we conducted our research in the context of a microfinance institution in Northern Vietnam. We suggest that the relevance of women’s relationships in their empowerment is likely true for women from most countries adhering to an ecology of embedded interdependence and perhaps universally. However, future research is needed to investigate these claims cross‐culturally (for a discussion on cross‐cultural psychology in developmental aid effectiveness research, see Brouwers, 2018; Chaudhary, 2018).

To conclude, to disentangle the directionality and dynamics of a relational perspective on women’s empowerment, future research is needed which follows young women from their adolescence until they are living together with their romantic partners. This research would offer the opportunity to investigate the development of personal empowerment and relational dynamics (i.e., IPV), which may affect women’s position within the household and beyond in society. To draw causal conclusions, this research would need to also investigate the impact of structural factors (i.e., access to resources) and cultural norms (i.e., gender norms).

### Practical implications

Our research set out to illustrate the importance of taking a relational perspective in understanding women’s empowerment. Based on our own and other research, we suggest two practical implications. First, women are not individual agents; they are embedded in social relationships, which influence their behaviour and define who they are (e.g., Fiske, 1992). This is perhaps even more relevant in countries in the Global South where the relational context in which women live is central to their personhood (for a similar argument, see Estrada‐Villalta & Adams, 2018). We suggest that interventions to strengthen women’s empowerment by solely focusing on strengthening women’s personal capacities (e.g., agency) may be not be that effective. Our research suggests that personal empowerment seems to be an important starting point, which interventions should stimulate. However, at the same time, they should focus on the relational context and power barriers, which may inhibit women (see Connell, 1987). Gender norms and power barriers strongly influence the meaning and development of women’s empowerment (e.g., Ibrahim & Alkire, 2007; Huis *et al.*, 2017). For example, women tend to internalize restrictive gender norms and justify IPV in Vietnam (Duvvury *et al.*, 2012). Interventions aiming to empower women need to understand and target gender norms, which may otherwise inhibit women and keep the power barriers in place.

Second, to achieve social change towards more egalitarian gender relations within the household, both parties of the relationship may need to be involved (see also Chowdhury & Patnaik, 2010). Previous research shows that men may feel excluded if interventions only target women and may respond by displaying power (i.e., emotional or physical). Thus, involving men may help to overcome relational friction (e.g., Rahman *et al.*, 2011). Recent research shows that women entrepreneurs in Sri Lanka and their husbands both need to learn to set goals with respect to their income generating activity to strengthen women’s position (e.g., Huis, Lensink, *et al*., 2019). In addition, other research shows that men and women need to be involved when deciding about family planning to change existing patterns while preserving women’s autonomy (Petterson & Sutton, 2017).

### Conclusion

To conclude, we suggest that women are not individual agents but are embedded in social relations, especially in more collectivist cultures. Women entrepreneurs have a say in financial expenditures in domains traditionally assigned to them (e.g., food, clothing). However, their power to have a say in larger expenditures such as business investments may be hampered by their experience of IPV. These decisions are conventionally not made by women but would be most indicative of greater empowerment. Interventions aiming to strengthen women’s position in their family and society could profit from a relational perspective by, for example, involving husbands or focusing on social norms to change gender roles towards more gender equity. We hope that our research encourages future research to consider the relational context in which women are embedded when striving to achieve the fifth development goal of the United Nations.

## Conflicts of interest

All authors declare no conflict of interest.

## Supporting information


**Appendix S1.** Supplementary materials.
