# Supplementary material for: A relational perspective on women’s empowerment: Intimate partner violence and empowerment among women entrepreneurs in Vietnam
Source: Br J Soc Psychol. 2019 Oct 30;59(2):365–86. doi: 10.1111/bjso.12348 (PMC7187353; doi:10.1111/bjso.12348)
Supplement: Supplementary file 1 — Appendix S1. Supplementary materials. [file BJSO-59-365-s001.docx]

**Online Appendix**

Table A1

*Items and subscales measuring intra-household decision-making at time 1 and time 2.*

|  | Time 1 | | Time 2 | |
| --- | --- | --- | --- | --- |
| Overall intra-household decision-making | component 1 | component 2 | component 1 | component 2 |
| *Larger expenditure decision-making* |  | |  | |
| Who makes most decisions about asking for a loan? | .226 | .163 | .316 | -.286 |
| Who makes most decisions about consumer durable items?   (TV,fridge, tape recorder etc.) | .310 | .194 | .354 | -.224 |
| Who makes most decisions about what health expenditures to  make? | .292 | -.247 | .281 | .212 |
| Who makes most decisions about saving for business and for  household? | .320 | .072 | .327 | .037 |
| Who makes most decisions about expenses for home purchase,  improvement or repair? | .281 | .232 | .368 | -.202 |
| Who makes decisions about where to invest surplus money? | .330 | .319 | .357 | -.035 |
| Who makes decisions about how to assist family members? | .264 | .359 | .367 | -.238 |
| Who makes most decisions about saving for household? | .304 | .281 | .310 | .042 |
| *Small expenditure decision-making* |  | |  | |
| Who makes most decisions about what food items to purchase? | .327 | -.426 | .196 | .472 |
| Who makes most decisions about what educational expenditures  to make (e.g., tuition)? | .321 | -.375 | .173 | .475 |
| Who makes most decisions about what clothing items to  purchase? | .327 | -428 | .163 | .523 |
| **Eigenvalues** | 3.79 | 1.73 | 3.68 | 1.81 |
| **% of variance** | 34.32 | 15.70 | 36.84 | 18.10 |


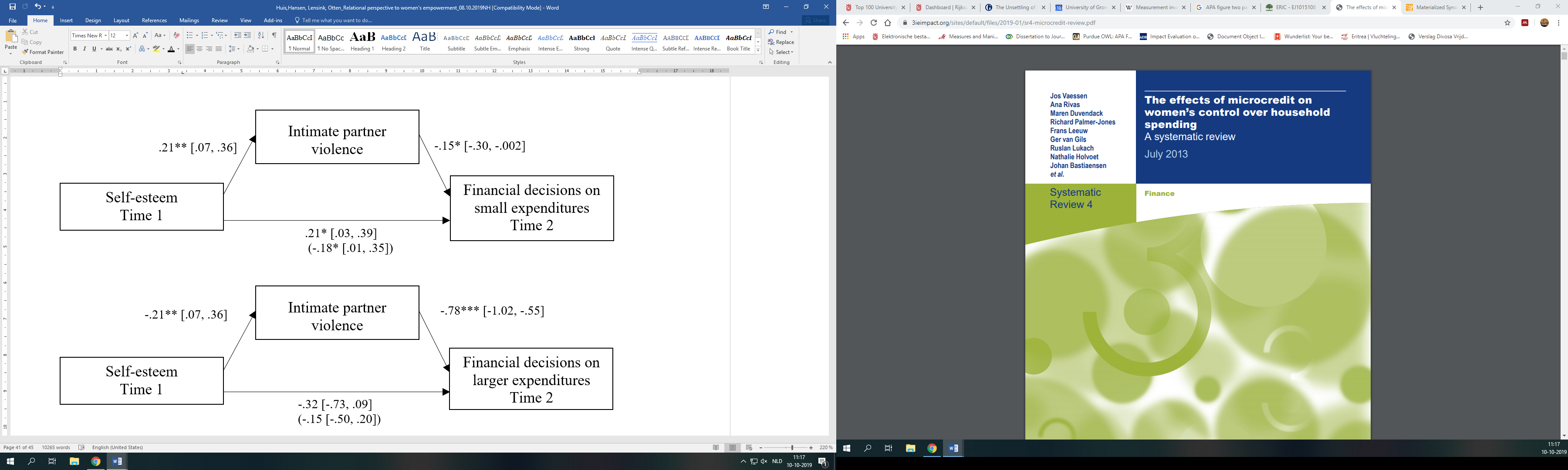


*Figure A1.* Standardized regression coefficients for the relationship between self-esteem at time 1 and intra-household decision-making on small (top) and larger expenditures (bottom) at time 2 as mediated by intimate partner violence at time 2. The standardized regression coefficients between self-esteem at time 1 and decision-making at time 2 are in parentheses. The confidence intervals for all coefficients are in box brackets. The indirect effect of self-esteem at time 1 via IPV at time 2 on small expenditures at time 2 is not significant -.03 [-.07, .001]. The indirect effect of self-esteem at time 1 via IPV at time 2 on larger expenditures at time 2 is significant -.17 [-.29, -.04]. * *p* < .05, ** *p* < .01, *** *p* < .001.


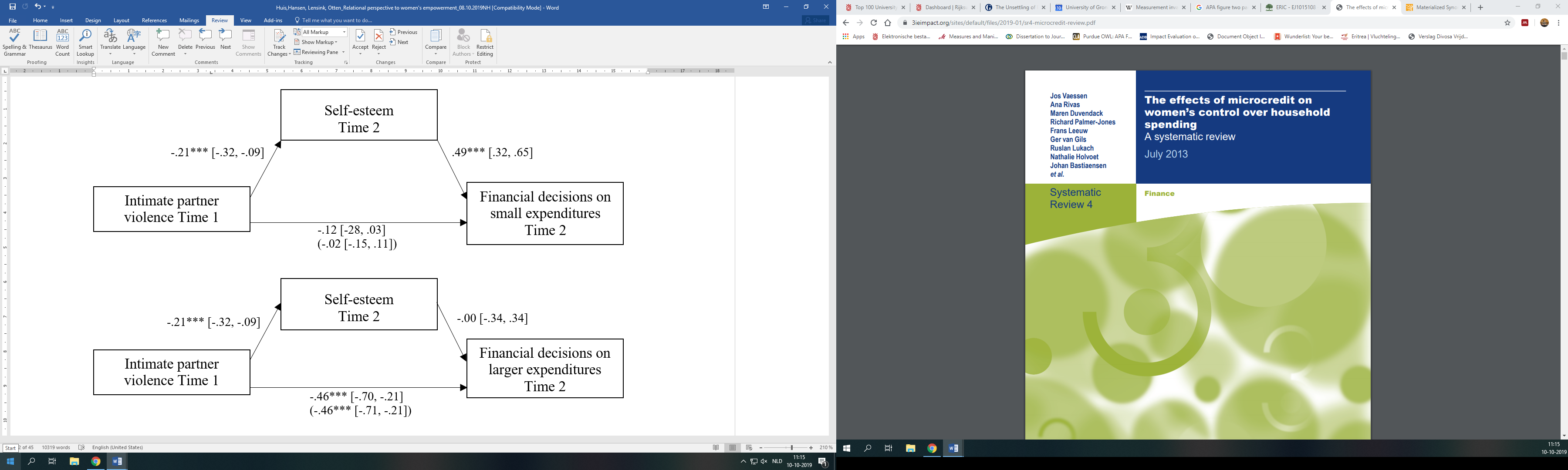


*Figure A2.* Standardized regression coefficients for the relationship between intimate partner violence at time 1 and intra-household decision-making on small (top) and larger expenditures (bottom) at time 2 as mediated by self-esteem at time 2. The standardized regression coefficients between intimate partner violence time 1 and decision-making time 2 are in parentheses. The confidence intervals for all coefficients are in box brackets. The indirect effect of IPV at time 1 via self-esteem at time 2 on small expenditures at time 2 is significant --.10 [-.18, -.03]. The indirect effect of IPV at time 1 via self-esteem at time 2 on larger expenditures at time 2 is not significant .00 [-.07, .07]. * *p* < .05, ** *p* < .01, *** *p* < .001.


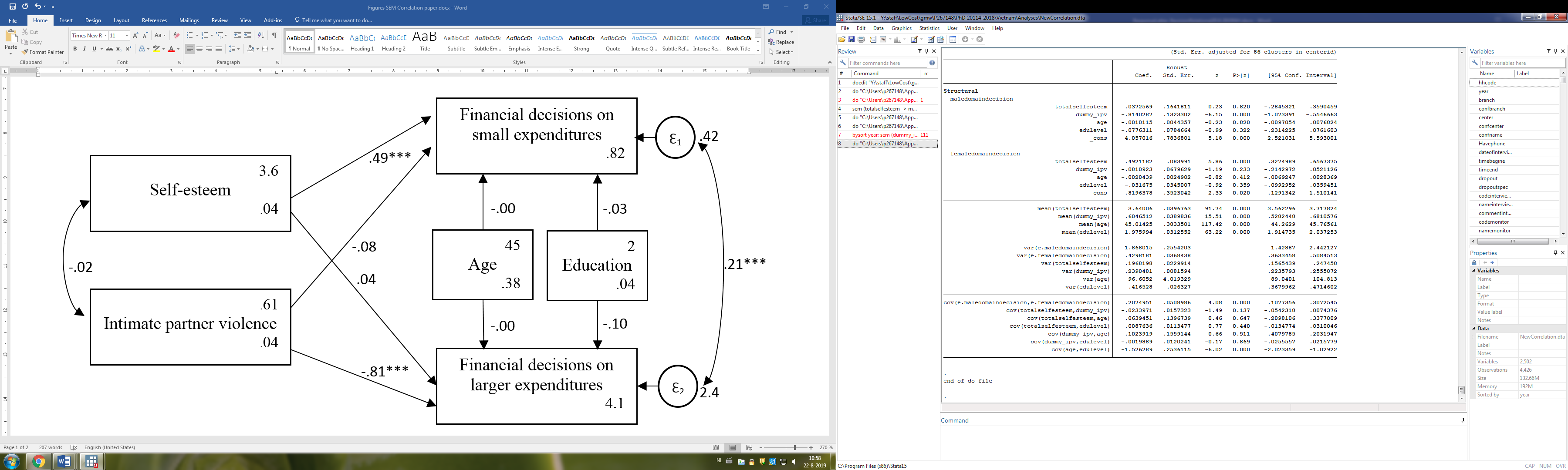


*Figure A3.* Conceptual model predicting women’s financial decision-making on small and larger expenditures with added control variables age and education at time 2. Means and robust standard errors are reported for the endogenous and control variables. SRMR <.001, CD = .175. * *p* < .05, ** *p* < .01, *** *p* < .001.

**Supplementary Material**

**Measurement invariance**

We tested measurement invariance for all study variables. However, important to note we only expected it for self-esteem and decision-making on small expenditures. Measurement invariance for each scale was assessed by multiple group confirmatory analysis, using the measurementInvariance() function of the semTools package in R. We used the criteria of CFI > .90, RMSEA ≤ .08, TLI ≥ .90, and SRMR < .10 as cut-off points for sufficient model fit (see also Vandenberg & Lance, 2000).

Unexpectedly, self-esteem did not show configural measurement invariance: χ^2^(70) = 2997.60, *p* < .001, CFI = .683, TLI = .593, RMSEA = .176, 90% CI [.162, .186], SRMR = .115. As expected, women’s decision-making on small expenditures was measurement invariant across time-points. Indices of model fit for configural invariance were acceptable: χ^2^(6) = 175.59, *p* < .001, CFI = 1.00, TLI = .100, RMSEA = .000, 90% CI [.000, .000], SRMR = .000, as were the indices of model fit for metric invariance: : χ^2^(2) = 166.49, *p* < .001, CFI = .998, TLI = .993, RMSEA = .042, 90% CI [.009, .079], SRMR = .014, and as were the indices of model fit for scalar invariance: : χ^2^(4) = 165.06, *p* < .001, CFI = .992, TLI = .988, RMSEA = .056, 90% CI [.034, .081], SRMR = .022.

With respect to the dummy-coded IPV variable and the decision-making on large expenditure we expected that women should differ which items they would experience at time 1 and time 2 (due to nominal structure of the response options). For example, women may experience another act of IPV or have gained a say over financial decisions on loans. Thus, we did not expect measurement invariance. As expected, women’s decision-making on larger expenditures showed no configural measurement invariance: χ^2^(40) = 1360.20, *p* < .001, CFI = .768, TLI = .675, RMSEA = .158, 90% CI [.151, .165], SRMR = .115. As expected, women’s experienced IPV showed no configural measurement invariance: χ^2^(28) = 921.33, *p* < .001, CFI = .691, TLI = .536, RMSEA = .158, 90% CI [.149, .166], SRMR = .076.
